# How drug resistance takes shape

**DOI:** 10.7554/eLife.14973

**Published:** 2016-03-24

**Authors:** Rinath Jeselsohn, Myles Brown

**Affiliations:** 1 Center for Functional Cancer Epigenetics and the Department of Medical Oncology, Dana-Farber Cancer Institute, Boston, United States; 1 Center for Functional Cancer Epigenetics and the Department of Medical Oncology, Dana-Farber Cancer Institute, Boston, United Statesmyles_brown@dfci.harvard.edu; 2 Department of Medicine, Harvard Medical School, Boston, United States; 2 Department of Medicine, Harvard Medical School, Boston, United States

**Keywords:** breast cancer, acquired drug resistance, somatic mutation, estrogen receptor alpha, hormone, selective estrogen receptor modulators, Human

## Abstract

Mutations in a hormone receptor can lead to therapeutic resistance by making it less able to bind and respond to hormone blocking drugs and by making it active, even when the hormome is not present.

**Related research article** Fanning SW, Mayne CG, Dharmarajan V, Carlson KE, Martin TA, Novick SJ, Toy W, Green B, Panchamukhi S, Katzenellenbogen BS, Tajkhorshid E, Griffin PR, Shen Y, Chandarlapaty S, Katzenellenbogen JA, Greene GL. 2016. Estrogen receptor alpha somatic mutations Y537S and D538G confer breast cancer endocrine resistance by stabilizing the activating function-2 binding conformation. *eLife* **5**:e12792. doi: 10.7554/eLife.12792

**Image** Estrogen (pink), followed by a co-activator (green), bind to estrogen receptor alpha (purple) in order to activate it

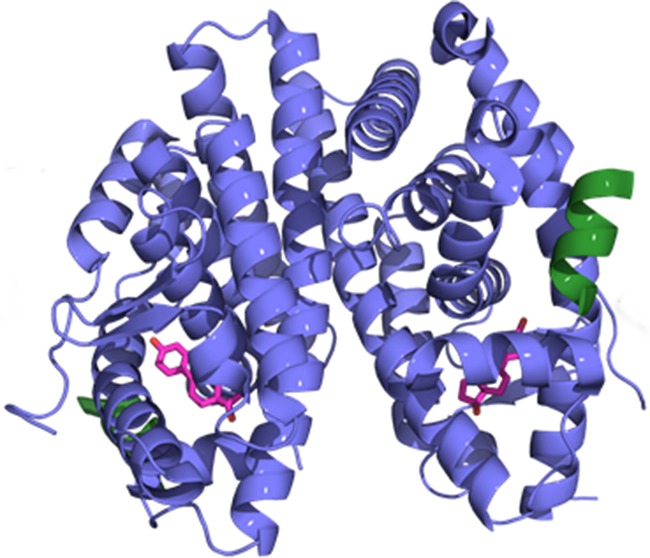

The majority of breast cancers are hormone receptor positive and are sensitive to the steroid hormone estrogen. This hormone binds to and activates a transcription factor known as estrogen receptor alpha (ERα), and drugs that reduce the production of estrogen or directly block estrogen binding by ERα can halt the growth of estrogen-sensitive cancers. However, in many cases, these endocrine therapies eventually stop working because the cancer cells develop resistance to the drugs. Recently, this resistance has been linked to a number of mutations in the gene for ERα that are almost exclusively found in patients who have received hormone therapy. The two most prevalent mutations to have been identified are the missense mutations Y537S and D538G. (Toy et al., 2013; Merenbakh-Lamin et al., 2013; Robinson et al., 2013; Jeselsohn et al., 2014 ).

The ERα mutations affect the part of the protein called the ligand-binding domain. Studies in breast cancer cell lines show that these mutations result in ERα activity, even in the absence of estrogen, and that they cause relative resistance to tamoxifen and other drugs that directly target ERα. Now, in eLife, Geoffrey Greene of the University of Chicago and colleagues – including Sean Fanning, Christopher Mayne and Venkatasubramanian Dharmarajan as joint first authors – report new insights into the effects that the two mutations have on the structure of ERα (Fanning et al., 2015). They also explore how these mutations can lead to drug resistance.

The ligand-binding domain of ERα consists of three layers of α–helices. The final helix, known as helix-12, acts as a molecular switch that changes position when estrogen binds to the domain. This enables co-activator proteins, such as the steroid receptor coactivator 3 (SRC3), to bind to and activate ERα. A previous study found that the ligand-binding domain from an ERα mutant known as Y537S has a structure that strongly resembles the wild-type domain when it is bound to estrogen (Nettles et al., 2008). This mutation stabilizes helix-12 in a conformation that allows the coactivator to bind (even in the absence of estrogen) by forming a hydrogen bond with another amino acid in ERα.

The crystal structure of the D538G mutant remained unknown and molecular modeling provided limited insight as to how this mutation allowed ERα to be active in the absence of estrogen. Furthermore, it was not clear how any of the mutations render cancer cells more resistant to drugs that target ERα. Some researchers proposed that the drug resistance was caused by decreased binding affinity to the ERα mutants, but few studies have been carried out to test this hypothesis.

Fanning et al. – who are based at Chicago, the University of Illinois at Urbana-Champaign, the Scripps Research Institute, the Memorial Sloan Kettering Cancer Center and Texas A&M University – addressed these questions by performing comprehensive biophysical and crystallographic studies on the Y537S and D538G mutations. This information is critical for the development of new molecules that target these mutations.

Fanning et al. show that SRC3 only binds to wild-type ERα in the presence of estrogen, while the Y537S and D538G mutants are able to recruit SRC3 in the absence of the hormone. In addition, ligand-binding assays demonstrate that the affinity for tamoxifen-mutant binding is lower than the affinity for tamoxifen-wild type binding. Taken together, the mutations stabilize the active form of ERα and make it harder for anti-cancer drugs to bind to it. However, the addition of estrogen still increased the ability of SRC3 to bind to both mutants, which suggests that depriving tumors of estrogen may still be important in treating cancers with these mutations.

Next, Fanning et al. obtained X-ray crystal structures of the ligand-binding domain from the D538G mutant on its own, and when it was bound to estrogen or tamoxifen. As expected, helix-12 in this mutant was more stable in the conformation that allows SRC3 to bind even in the absence of estrogen. This stability, however, is structurally different from that observed in the Y537S mutant and is more subtle. In addition, in the absence of estrogen, SRC3 binds to the Y537S mutant with an affinity that is higher than when it binds to the D538G mutant. These differences between the two mutations may have clinical implications, as recent data suggests that they may be associated with different outcomes in cancer patients (Chandarlapaty, 2015). However, additional studies are needed to fully understand how important these differences are.

In the presence of tamoxifen, the ligand-binding domain of the D538G mutant adopts a conformation that is different to that of the wild type domain. This difference is mainly due to changes in a loop that connects helix-11 and helix-12, and leads to a decrease in the inhibitory activity of tamoxifen. Likewise, computational modeling suggests that the Y537S mutation also induces a conformational change in the ligand-binding domain that reduces the ability of tamoxifen to inhibit ERα activity. Thus, resistance to tamoxifen stems from a combination of the drug being less likely to bind to the mutant domains and being less effective when it does bind.

The work of Fanning et al. significantly advances our understanding of the link between structural alterations of the ERα mutant and drug resistance, suggesting that new drugs will be needed to overcome the resistance caused by these mutations. These new drugs will need to bind to the mutant domains with higher affinity than existing drugs and be able to either stabilize helix-12 in the conformation that prevents SRC3 or other co-activators from binding, or promote the degradation of ERα. Future studies will also need to focus on the mutation known as E380Q that is also detected in breast cancer patients with resistant disease and may have similar effects to the mutations studied by Fanning et al.
